# Supplementary material for: A Global Screen for Assembly State Changes of the Mitotic Proteome by SEC-SWATH-MS
Source: Cell Syst. 2020 Feb 26;10(2):133–155.e6. doi: 10.1016/j.cels.2020.01.001 (PMC7042714; doi:10.1016/j.cels.2020.01.001)

Q9H2H9 | S38A1_HUMAN | SLC38A1 ATA1 NAT2 SAT1 SNAT1
Monomer MW [kDa]: 54.048 Monomer expected elution fraction: 46

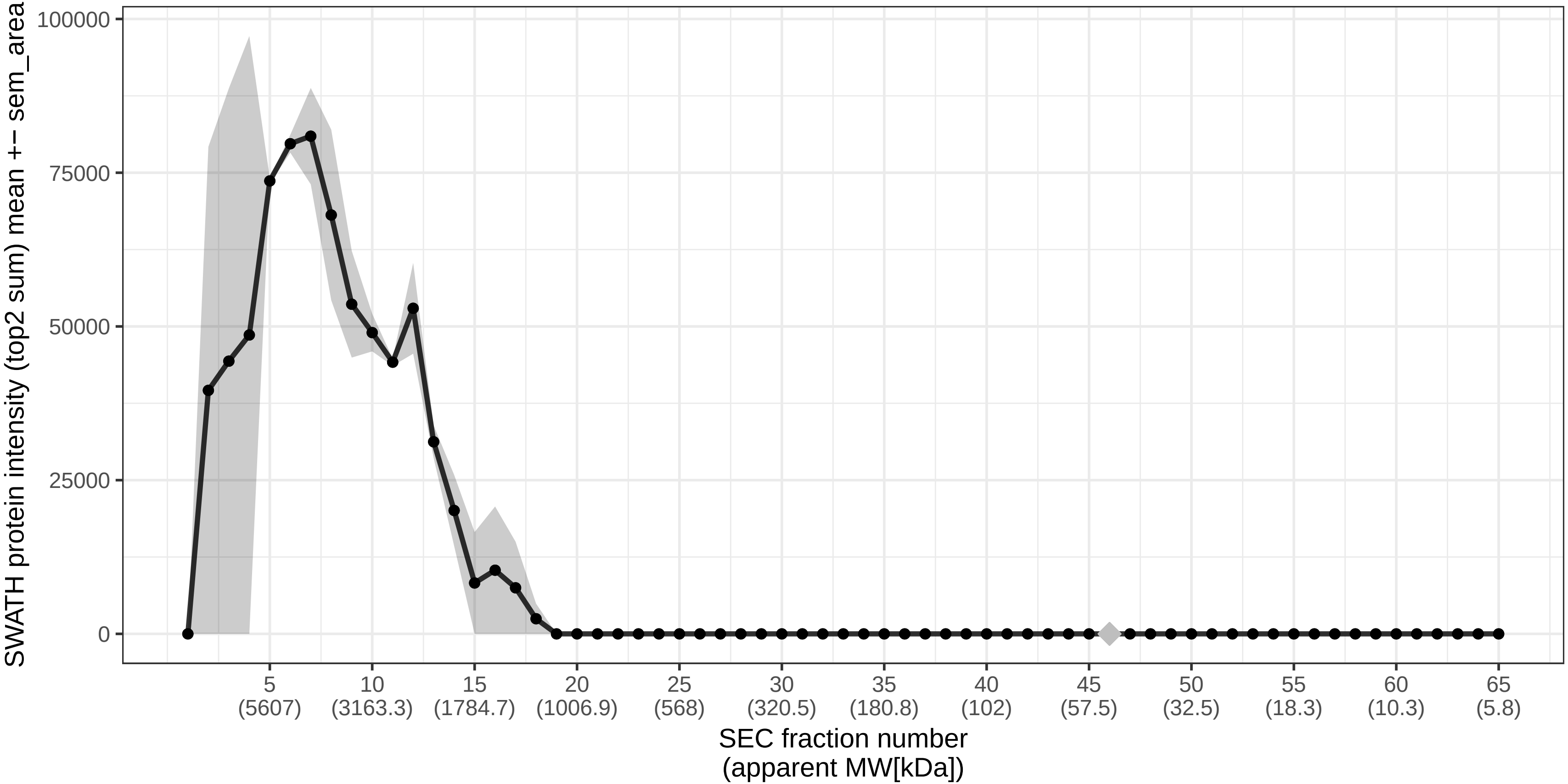

Supplement: Data S1. SEC-SWATH-MS Protein Chromatograms, Related to Figure 1 [file mmc6.zip › SECchrom_Q9H2H9_S38A1_HUMAN_SLC38A1_ATA1_NAT2_SAT1_SNAT1.pdf]
